# Groin pain aggravated in short term contracted by COVID-19 in THA patients: a case-crossover study

**DOI:** 10.1186/s13018-024-04862-1

**Published:** 2024-06-24

**Authors:** Hongjie Chen, Peng Lai, Haiming Lu, Jun Zhu, Weilin Sang, Cong Wang, Yiming Zhong, Libo Zhu, Jinzhong Ma

**Affiliations:** 1 Department of Orthopedics, Shanghai General Hospital, Shanghai Jiao Tong University School of Medicine, Shanghai, 200080 China; 2 Department of Neurosurgery, Shanghai General Hospital, Shanghai Jiao Tong University School of Medicine, Shanghai, 201620 China; 3 Department of Orthopedics, Peking University Third Hospital, Beijing, 100191 China

**Keywords:** Groin pain, COVID-19, Total hip arthroplasty (THA), Case-crossover study

## Abstract

**Background:**

The coronavirus disease 2019 (COVID-19) rapidly spreads worldwide and causes more suffering. The relation about the aggravation of inguinal pain and COVID-19 was unclear in patients with total hip arthroplasty (THA). This study aimed to evaluate the risk of groin pain aggravation in short-term THA patients after COVID-19.

**Methods:**

Between 2020 and 2022, 129 patients with THA who were affected COVID-19 were enrolled. A short-standardized questionnaire was administered during follow-up to inquire about the aggravation of groin ache before and after SARS-COV-2 affection. Furthermore, we evaluated the potential association between the presence of increased pain and various factors, including age, gender, body mass index, diagnosis, and length of hospital stay.

**Results:**

The case-crossover study revealed an increased risk of inguinal soreness aggravation when comparing 8 weeks after COVID-19 with 12 weeks before COVID-19 (Relative risk [RR], 9.5; 95% Confidence intervals [CI], 2.259–39.954). For COVID-19 positive patients, multivariate analysis showed length of stay was an independent factor significantly associated with increased risk of aggravation of groin pain (Odds ratio [OR], 1.26; 95%CI, 1.03–1.55, *p* = 0.027).

**Conclusion:**

This study confirms the association between COVID-19 and the exacerbation of soreness in the groin region in THA patients and extended length of stay is a possible contributing factor. This study expands the current literature by investigating the risk of aggravation of inguinal pain in patients with THA after COVID-19, providing valuable insights into postoperative outcomes in this specific population.

*Trial registration* This retrospective study was approved by the Institutional Review Board of Shanghai general hospital (No.2023-264).

**Supplementary Information:**

The online version contains supplementary material available at 10.1186/s13018-024-04862-1.

## Introduction

Total hip arthroplasty (THA) is a reliable procedure that effectively reduces pain, improves function, and enhances the quality of life for patients with advanced hip disease [1, 2]. Over the past 2–3 decades, the number of arthroplasty procedures has increased, and is projected to rise by 176% by 2040 and 659% by 2060 in THA procedures [3–5]. Although primary THA has been voted the most successful operation of the century, more than 10% of patients report postoperative dissatisfaction [6–8]. Postoperative pain is one of significant reasons for dissatisfaction, which affects up to 18.3% of patients at 3 months and 12.9% at 24 months of follow-up [9]. The most common causes include infection, aseptic loosening of the acetabular component, iliopsoas tendonitis, impingement, synovitis due to metal or polyethylene debris [10]. This pain can lead to immobility, contributing to various medical complications [11], such as venous thrombosis and pneumonia, with potentially serious health consequences.

To make matters worse, SARS-COV-2 infection may cause more disturbing for THA patients [12–14]. Notably, the prevalence of arthralgia or myalgia during the COVID-19 pandemic was reported to be 15.5% within the first 5 months [15], while new-onset chronic pain (> 3 months) affected 19.6% of COVID-19 patients [16]. SARS-COV-2, the virus responsible for COVID-19, primarily invades the respiratory system and triggers a systemic inflammatory response characterized by upregulated inflammatory cells and factors [17], which induces muscle and joint pain [18].

Studies have shown that THA patients have higher levels of inflammatory cells and cytokine in the periprosthetic tissues than normal [19], such as tissue damage from surgery and chronic inflammation from wear debris [20]. Thus, the inflammatory response following SARS-COV-2 infection may be the stronger or earlier to occur in the THA patients than the healthy [21], with a higher likelihood of pain occurring [22]. In turn, the inflammatory storm caused by SARS-COV-2 infection further inhibits the repair of periprosthetic tissues, adversely affecting THA patients [22]. Hence, the phenomenon is all the more worthy of our attention.

Previous studies have confirmed COVID-19 causes pain, such as arthralgia or myalgia, but whether it increases groin pain in short-term THA patients has not been explored. Therefore, this study aimed to investigate the association between COVID-19 and increased pain in inguinal area in THA patients. By addressing this research gap, we aim to provide valuable insights for clinical practice.

## Methods

### Patient selection

Patients who underwent primary THA between August 5, 2020, and September 23, 2022, were reviewed. All THAs were performed by the same surgeon group in the center with uncemented implants. In accordance with the standard, a rehabilitation physician will perform hip flexion and extension exercises as well as muscle strength restoration training for the patient 24 h after the operation. And after 72 h, the patient will be assisted to go down to the ground with the aid of a walker under the guidance of the rehabilitation physician. The following inclusion criteria were applied: (1) all patients tested negative for COVID-19 before THA, as confirmed by polymerase chain reaction (PCR) test and medical history; (2) patients had indications for hip arthroplasty other than malignancy (osteoarthritis, development dysplasia, and femoral neck fracture); (3) patients had no inflammatory disease or immunosuppression; (4) patients were infected with SARS-COV-2 after a 12-week recovery period after THA;

### Data collection

Patient characteristics, including age, gender, height, weight, body mass index (BMI), the surgical approach for THA, length of stay for THA, comorbidities, complications and X-rays were collected from the electronic patient medical record system of hospital. A short-standardized questionnaire (supplymentary 1) was administered by specialized doctor via phone interview to gather information on the incidence of COVID-19 and the occurrence of groin ache aggravation. The treatment for COVID-19 (such as inhaled steroids or anti-virals drug) and treatment outcomes (weather become septic or admitted to ICU or die) were also recoreded. To rule out the effects of surgical trauma, we included the occurrence of aggravation of pain from 3 months after THA. The timing and duration of pain aggravation until it returned to baseline levels were recorded. COVID-19 was defined as a positive PCR test or antigen test for SARS-COV-2, and the date of COVID-19 positivity was defined as the day of a positive test. The COVID-19 tests are conducted by qualified institutions and subjects can query the results in the next day.

### Study design

In case cross-over study, the time of positive test was set as time zero and the study was divided in two times frames: 12 weeks before time zero to time zero (control period) and time zero to 8 weeks after time zero (risk period). The 8-week time window was chosen as a short period to observe immediate risks following infection [23]. When the patient was included, detailed questions about the pain was asked by standardized questionnaire and the occurrence of pain aggravation in COVID-19 positive patients was calculated in the two times frames. The final follow-up was conducted on May 1, 2023.

### Statistical analysis

Statistical analyses were performed using the Statistical software (IBM, Armonk, NY). The McNemar test was used to investigate the differences in categorical variables (pain aggravation or not), and the chi-square test was applied to calculate the relative risk (RR) with 95% confidence intervals (95% CI) in the frequencies of pain aggravation by comparing the 8-week period after COVID-19 (risk period) with the 12-week period before COVID-19 (control period). Risk factors for increased pain in COVID-19 patients were evaluated using univariate analysis with the occurrence of pain aggravation as the dependent variable. For categorical variables, χ^2^ test was used. For continuous variables, the t-test is used for data that conforms to a normal distribution, and the non-parametric test is used for non-normally distributed data. In addition, age, head size, cup size, length of stay and days after THA were included in multivariate logistic regression models based on univariate analyses with *p* < 0.1 and clinical experience, and odds ratios (OR) and respective 95% confidence intervals (CI) were calculated using the R (version 4.3.0) with R Studio (version 2023.03.1 + 446). The cut-off for statistical significance was set at *p* < 0.05.

## Results

### Patient characteristics

**Fig. 1:**
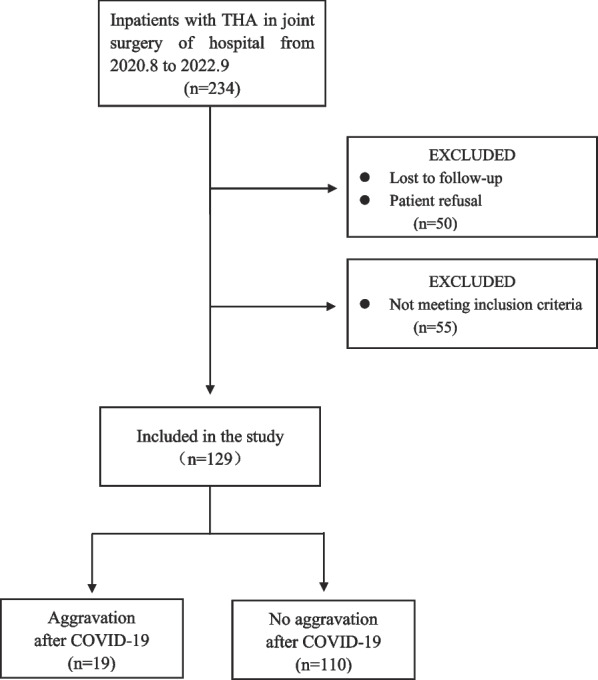
Flow diagram

The flow diagram was shown in Fig. 1. One hundred and twenty-nine patients were registered as positive for COVID-19 (Table 1) during the study period. Of those, 44 (34.1%) were men and the mean age was 63.7 years (standard deviation [SD], 13.8). The media of body mass index (BMI) were 23.9 kg/m^2^ (interquartile range [IQR], 21.9–26.9). And 119 THAs (92.2%) were performed using the direict anterior approach (DAA), with a median length of stay of 8 days (IQR,7–10). The 106 patients (82.2%) were treated only with symptomatic treatment (including physical cooling, NSAID or cough expectorant drugs), and the rest were combined with antiviral therapy (inclding ribavirin, molnupiravir, lopinavir, ritonavir, favipiravir, etc.). None had received treatment with hormones or immunosuppressants. None of the patients affected by COVID-19 experienced sepsis, ICU, or death. Characteristics of increased groin pain in short-term THA patients are shown in Table 2. For the two patients who experienced increased pain prior to infection, they suffered for 78 days and 238 days, respectively, and had not yet recovered. All patients with increased pain had no abnormality after X-ray review.

**Table 1:** Baseline characteristics of the COVID-19 positive patients

Characteristics	COVID-19 positive patients(n = 129)
Men, n, (%)	44 (34.1%)
Age, years, mean (SD)	63.7 (13.8)
Height, cm, median (SD)	159.4 (8.3)
Weight, kg, mean (SD)	62.3 (12.2)
BMI, kg/cm^2^, median [IQR]	23.9 [21.9, 26.9]
Clinical characteristics	
Hypertension, n, (%)	37 (28.9%)
Diabetes, n, (%)	46 (35.7%)
ASA score	2 [1, 2]
Diagnosis, n, (%)	
Degenerative joint disease	79 (61.2%)
DDH	17(13.2%)
Necrosis of the femoral head	17(13.2%)
Femoral neck fracture	12 (9.3%)
Others	4 (3.1%)
Approach, n, (%)	
DAA	119 (92.2%)
PLA	10 (7.8%)
Head size, cm, median [IQR]	28 [28, 32]
Cup size, cm, median [IQR]	46 [46, 50]
Length of stay, days, median [IQR]	8 [7, 10]
Follow-up	
Days after THA, days, median [IQR]	578 [405, 808]
Dislocation, n, (%)	5 (3.9%)
Periprosthetic fracture, n, (%)	1 (0.8%)
Treatment for COVID-19	
Symptomatic treatment	106(82.2%)
Symptomatic and antiviral treatment	23(17.8%)

*COVID-19* Coronavirus disease 2019; *BMI* Body mass index; *ASA* American Society of Anesthesiologists; *THA* Total hip arthroplasty; *DAA* Direct anterior approach; *PLA* Posterolateral approach; *IQR* Interquartile range; *SD* Standard deviation

**Table 2:** Characteristics of increased groin pain in short-term THA patients

Characteristics	Patients with increased groin pain	
	After COVID-19 (n = 19)	Before COVID-19 (n = 2)
Pain duration, days; median [IQR]	21 [8–54]	78、238
Complete relief, n, (%)	11(58%)	/
Partly relief, n, (%)	3 (16%)	/
No relief, n, (%)	5 (26%)	2 (100%)

*COVID-19* Coronavirus disease 2019; *THA* Total hip arthroplasty; *IQR* Interquartile range

### Risk of groin pain aggravation in patients with THA after SARS-COV-2 infection

In the case-crossover design, two patients (1.6%) experienced pain aggravation during the 12-week control period before COVID-19. Both of them suffered more serious pain in inguinal area during the 8-week risk period after COVID-19. Additionally, 17 patients (13%) who did not have pain aggravation before COVID-19 developed pain aggravation during the 8-week risk period after COVID-19. Significant differences in the incidence of pain aggravation were observed between risk period and control period (χ^2^ = 15.059, *p* < 0.0001) (Table 3). In risk period, 19 of 129 patients (14.7%) who were compared with two of 129 patients (1.6%) in the control period felt more pain with a significantly increased risk (RR, 9.5; 95%CI, 2.259–39.954; *p* < 0.001) (Table 4).

**Table 3:** Differences of pain aggravation between control period and risk period in COVID-19 positive patients

Case-crossover design	8 weeks after COVID-19(risk period)		
12 weeks before COVID-19(control period)	Aggravation(n)	No aggravation(n)	All(n)
Aggravation, n, (%)	2	0	2
No aggravation, n, (%)	17	110	127
All(n)	19	110	129

The McNemar test was used to investigate the differences in pain aggravation (yes or no) by comparing the 12-week period prior to the COVID-19 with the 8-week period after COVID-19 (risk period)

**Table 4:** Frequency of aggravation in groin pain before and after SARS-COV-2 infection

Case-crossover design	Aggravation/All	Relative risk(95%CI)
12 weeks before COVID-19(control period)	2/129(1.6%)	Reference
8 weeks after COVID-19(risk period)	19/129(14.7%)	9.5(2.259–39.954)

*CI* Confidence interval; *COVID-19* Coronavirus disease 2019

### Risk factors for pain aggravation in patients after SARS-COV-2 infection

**Fig. 2:**
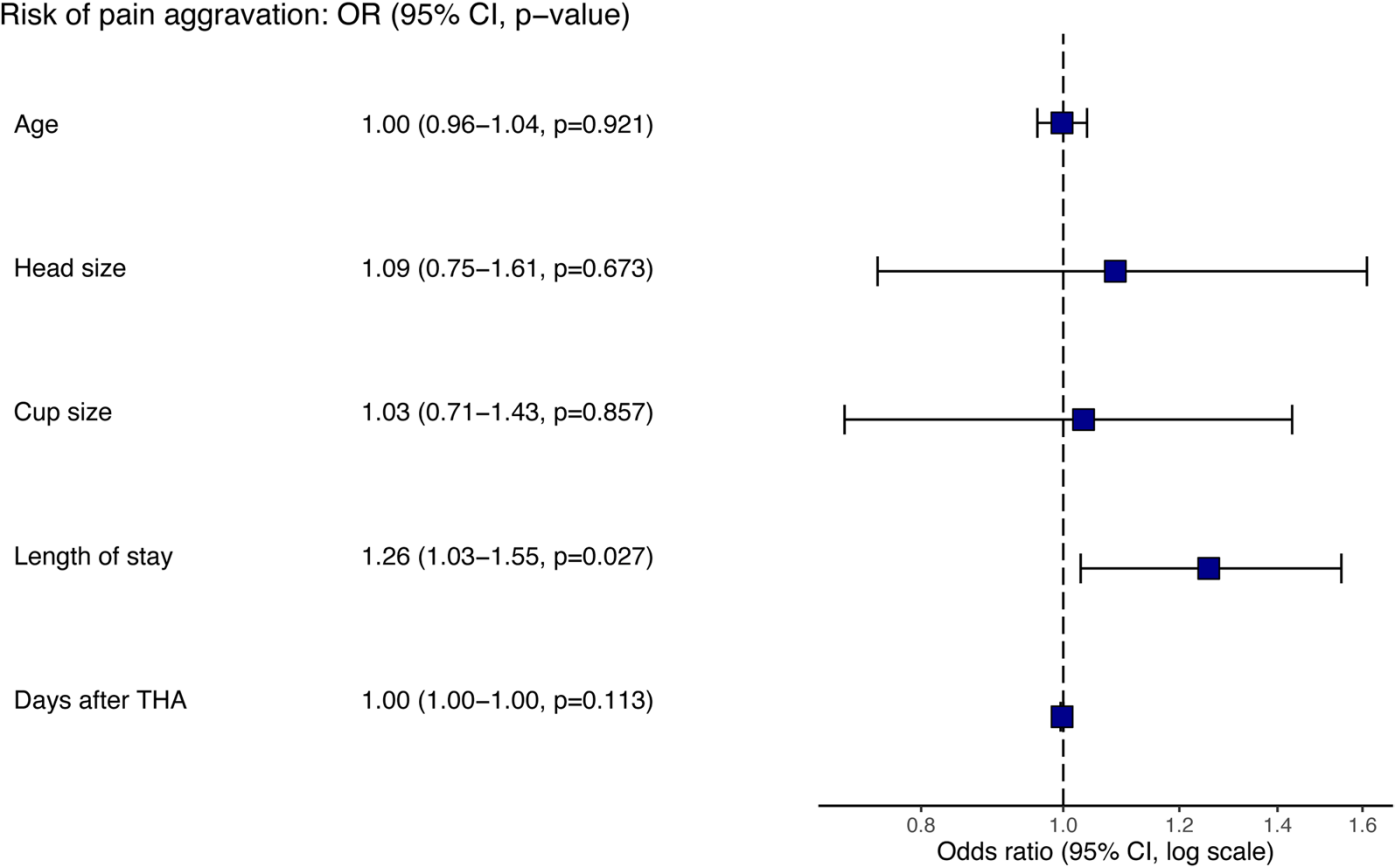
Forest plot. Odds ratios (OR) and 95% confidence intervals (CI) of factors of aggravation of groin pain in THA patients with COVID-19. Abbreviation: THA, total hip arthroplasty

The demographic and clinical features of patients with and without pain aggravation after COVID-19 are presented in Table 5. Univariate analysis showed there was no significant difference in all factors listed in the table between the groups of aggravation and no aggravation of pain after SARS-COV-2 infection. However, multivariate logistic regression analyses of possible risk factors for pain aggravation after COVID-19 revealed that the length of stay was an independent factor significantly associated with increased risk (OR 1.26; 95%CI, 1.03–1.55, *p* = 0.027) (Fig. 2).

**Table 5:** Comparison analysis of the factors on the aggravation of groin pain in patients with THA after COVID-19 positive

Characteristics	Aggravation after COVID-19(n = 19)	No aggravation after COVID-19(n = 110)	*p* value
Men, n, (%)	8 (42%)	37 (34%)	0.428
Age, years, mean (SD)	62.4(12)	63.9 (14)	0.670
Height, cm, median (SD)	159.6(9.2)	159.3(8.2)	0.899
Weight, kg, mean (SD)	63.6(15.0)	62.1(11.8)	0.637
BMI, kg/cm^2^, median [IQR]	24.7 [22.4, 28.3]	23.9 [21.9, 26.8]	0.537
Hypertension, n, (%)	3 (16%)	34 (31%)	0.189
Diabetes, n, (%)	6 (32%)	40 (36%)	0.688
ASA score, median [IQR]	2 [1, 2]	2 [1, 2]	0.763
Approach in THA			0.663
DAA, n, (%)	18 (95%)	101 (92%)	
PLA, n, (%)	1 (5%)	9 (8%)	
Length of stay, days, median [IQR]	8 [8, 13]	8 [7, 10]	0.056
Days after THA, days, median [IQR]	482 [336, 634]	613.5 [409, 813]	0.097
Head size, cm, median [IQR]	32 [28, 32]	28 [28, 32]	0.196
Cup size, cm, median [IQR]	48[46,50]	46[46,50]	0.234
Dislocation, n, (%)	1 (5%)	4 (4%)	0.736
Treatment			1.000
Symptomatic treatment	16(12.4%)	90(69.8%)	
Symptomatic and antiviral treatment	3(2.3%)	20(15.5%)	

*COVID-19* Coronavirus disease 2019; *BMI* Body mass index; *ASA* American Society of Anesthesiologists; *DDH* Developmental dysplasia of the hip; *THA* Total hip arthroplasty; *DAA* Direct anterior approach; *PLA* Posterolateral approach

## Discussion

The present retrospective study aimed to investigate the relationship between COVID-19 and the aggravation of inguinal ache in patients who underwent THA. The findings suggest that SARS-COV-2 infection increases the risk of groin pain during the eight weeks following infection. This indicates that the influence of COVID-19 on patients who underwent THA extends beyond the acute phase, as they continue to experience pain aggravation.

Pain is often associated with viral illnesses and is believed to result from the acute cytokine response, typically resolving after fever subsides [24]. However, Hoong observed that COVID-19 related arthralgia appears to differ from generalized body aches and myalgia commonly seen in viral infections [25], as it does not align with the typical prodromal symptoms. In our study, although the 19 patients (14.7%) with pain aggravation account for a small percentage of the total 129 COVID-19 patients, we observed that these patients were usually older (with the mean age of 62.4 years), experienced prolonged symptoms (with the median pain duration of 21 days). In addition, pain occurred a few days before or after the onset of fever and respiratory symptoms, which differs from commonly viral infections.

In our case-crossover study, we found that groin soreness aggravation occurred shortly after a positive COVID-19 test in most of the 19 patients, suggesting a potential causal relationship. Afterwards, we observed a significant increase in the frequency of pain aggravation during the 8 weeks after COVID-19 when compared to the 12 weeks before COVID-19. In addition, the incidence of aggravation of inguinal pain within 8 weeks after SARS-COV-2 affection was almost 10 times higher than before. These results indicated that COVID-19 played a vital role in the aggravation of groin pain. However, Maezawa et al. demonstrated that patients with THA did not change significantly in hip pain before and after COVID-19 pandemic [26]. In Maezawa’s research, the division of group was based on the different phases of SARS-COV-2 epidemics in the population, and it is not certain that all subjects during the pandemic were infected with COVID-19. The two studies differ markedly in the time-point settings and infection status of subjects, thus the results are probably different.

For COVID-19 positive patients, the length of stay was associated with an increased risk of pain aggravation in the multivariate analyses. The evidence is overwhelming that the number of comorbidities, the American Society of Anesthesiologists (ASA) score > 2, and the presence of cardiac or pulmonary disease are related to the length of stay [27]. However, the common comorbidities in this study including diabetes (*p* = 0.688) and hypertension (*p* = 0.189) were not significantly different between aggravation group and no aggravation group, and the same for ASA score (*p* = 0.763). Moveover, risk factors [28] such as age [29], high BMI, frailty, anemia, and blood transfusions were involved with the length of stay while age (*p* = 0.670) and BMI (*p* = 0.537) did not differ significantly in our study. Thus, it can be hypothesized that frailty and poor health increase the length of stay, which account for this phenomenon, as frail patients face more severe challenges after SARS-COV-2 infection [30, 31].

It has been reported in the literature that different types of prosthesis have an effect on the incidence of pain, with dual-mobility prosthesis having the lowest incidence of pain, fixed-bearing (FB) prosthesis following, and hip resurfacing having the highest incidence of pain [32–34]. Furthermore, the larger femoral heads have previously been shown to present a higher likelihood of causing inguinal soreness than smaller femoral heads [32]. In our study, the patients were using FB prosthesis and we found that the size of the head (*p* = 0.196) and cup (*p* = 0.234) of the FB prosthesis had no effect on inguinal pain.

Factors that influenced pain after surgery also included preoperative anxiety/depression symptoms [35], preoperative pain intensity [36], preoperative sleep quality [37], and lower patient participation in pain management [38], with the exception of the duration of hip arthroplasty [39] and enhanced recovery after surgery (ERAS) [40]. While the effect of gender and ASA score on pain is controversial [35, 36, 41], and our study showed the gender (*p* = 0.428) and ASA score (*p* = 0.763) had no influence on soreness in the groin region.

In most patients with increased pain, it was observed that soreness in inguinal area persisted for more than a week. Among the 19 patients who experienced increased pain after COVID-19, 11 of them were completely relieved of pain after the acute phase, while 3 patients did not experience relief. Among the remaining 5 patients, 3 patients reported increased pain in response to weather changes. Although more than half of the 19 patients had a short duration of pain and returned to baseline levels without requiring hospitalization, it should not be ignored that eight patients (42%) continued to suffer from pain, which significantly impacted their daily lives for a period of 4 months or more. This finding aligns with the emerging syndrome of post-acute COVID-19, also known as “long COVID”, in which symptoms may remain for a long time after a relatively minor COVID-19 infection has recovered [42, 43]. Furthermore, as patients with THA experience the long-term impact or an extended course of COVID-19, clinicians must be cognizant of this in the diagnosis and management of patients with a history of COVID-19 who present with groin pain and adequately distinguish it from other diseases.

This study has some limitations. Inguinal pain aggravation was assessed via a “yes/no” scale, as it was easier for elderly patients to understand and answer simple questions, although a pain severity score can provide more details. Furthermore, the retrospective case-crossover design introduces the possibility of recall bias and reduces the accuracy of data collection. However, the design has inherent strengths, such as the close proximity of the retrospective control period and the fact that each patient serves as their own control [44]. These features help minimize potential confounding factors and enhance the efficiency of the study. To improve upon our study, future research should consider employing a more comprehensive assessment of pain severity to capture the nuanced nature of groin ache. Additionally, efforts should be made to include a larger number of COVID-19 positive patients to enhance the generalizability of the findings. Incorporating multi-center collaborations or longitudinal studies could overcome these limitations and provide a more comprehensive understanding of the relationship between COVID-19 and inguinal soreness in patients with THA.

## Conclusions

The case-crossover study indicate that COVID-19 is associated with a significantly increased risk of aggravation of groin ache in THA patients during 8 weeks after COVID-19. Further research indicated that length of stay was associated with an increased risk of pain exacerbation. In summary, this study confirms the association between COVID-19 and the exacerbation of inguinal pain in THA patients. This study expands the current literature by investigating the risk of aggravation of groin pain in patients with THA after COVID-19, providing valuable insights into postoperative outcomes in this specific population.

### Supplementary Information

Below is the link to the electronic supplementary material.

Supplementary file 1: Short-standardized questionnaire about groin pain.

## Data Availability

Data available upon reasonable request.

## Abbreviations

COVID-19: Coronavirus disease 2019
THA: Total hip arthoplasty
RR: Relative risk
OR: Odds ratios
CI: Confidence intervals
DDH: Developmental dysplasia of the hip
DAA: Direct anterior approach
PLA: Posterolateral approach
PCR: Polymerase chain reaction
BMI: Body mass index
ASA: American society of anesthesiologists
SD: Standard deviation
IQR: Interquartile range

## References

[1] Mei XY, Gong YJ, Safir O (2019). Long-term outcomes of total hip arthroplasty in patients younger than 55 years: a systematic review of the contemporary literature. Can J Surg.

[2] Mackenzie JR, O'Connor GJ, Marshall DA (2012). Functional outcomes for 2 years comparing hip resurfacing and total hip arthroplasty. J Arthroplast.

[3] Shichman I, Roof M, Askew N (2023). Projections and epidemiology of primary hip and knee arthroplasty in medicare patients to 2040–2060. JB JS Open Access.

[4] Singh JA (2011). Epidemiology of knee and hip arthroplasty: a systematic review. Open Orthop J.

[5] Rupp M, Lau E, Kurtz SM (2020). Projections of primary TKA and THA in germany From 2016 through 2040. Clin Orthop Relat Res.

[6] Halawi MJ, Jongbloed W, Baron S (2019). Patient dissatisfaction after primary total joint arthroplasty: the patient perspective. J Arthroplast.

[7] Learmonth ID, Young C, Rorabeck C (2007). The operation of the century: total hip replacement. Lancet.

[8] Heath EL, Ackerman IN, Cashman K (2021). Patient-reported outcomes after hip and knee arthroplasty: results from a large national registry. Bone Jt Open.

[9] Lavigne M, Laffosse J-M, Ganapathi M (2011). Residual groin pain at a minimum of two years after metal-on-metal tha with a twenty-eight-millimeter femoral head, tha with a large-diameter femoral head, and hip resurfacing. JBJS.

[10] Henderson RA, Lachiewicz PF (2012). Groin pain after replacement of the hip. J Bone Joint Surg Br.

[11] Gaffney CJ, Pelt CE, Gililland JM (2017). Perioperative pain management in hip and knee arthroplasty. Orthop Clin North Am.

[12] Giordano L, Cipollaro L, Migliorini F (2021). Impact of Covid-19 on undergraduate and residency training. Surgeon.

[13] Migliorini F, Torsiello E, Spiezia F (2021). Association between HLA genotypes and COVID-19 susceptibility, severity and progression: a comprehensive review of the literature. Eur J Med Res.

[14] Migliorini F, Vaishya R, Eschweiler J (2022). Vitamins C and D and COVID-19 susceptibility, severity and progression: an evidence based systematic review. Medicina (Kaunas).

[15] Cipollaro L, Giordano L, Padulo J (2020). Musculoskeletal symptoms in SARS-CoV-2 (COVID-19) patients. J Orthop Surg Res.

[16] Soares FHC, Kubota GT, Fernandes AM (2021). Prevalence and characteristics of new-onset pain in COVID-19 survivours, a controlled study. Eur J Pain.

[17] Oronsky B, Larson C, Hammond TC (2023). A review of persistent post-COVID syndrome (PPCS). Clin Rev Allergy Immunol.

[18] Manjavachi MN, Motta EM, Marotta DM (2010). Mechanisms involved in IL-6-induced muscular mechanical hyperalgesia in mice. Pain.

[19] Louis Bridges S, Sun D, Graham ZA (2023). Muscle inflammation susceptibility: a potential phenotype for guiding precision rehabilitation after total hip arthroplasty in end-stage osteoarthritis. HSS J Musculoskelet J Hosp Special Surg.

[20] Costa MD, Donner S, Bertrand J (2023). Hypersensitivity and lymphocyte activation after total hip arthroplasty. Die Orthopädie.

[21] del Valle DM, Kim-Schulze S, Huang HH (2020). An inflammatory cytokine signature predicts COVID-19 severity and survival. Nat Med.

[22] Schett G, Manger B, Simon D (2020). COVID-19 revisiting inflammatory pathways of arthritis. Nat Rev Rheumatol.

[23] Nalbandian A, Sehgal K, Gupta A (2021). Post-acute COVID-19 syndrome. Nat Med.

[24] Kelvin AA, Banner D, Silvi G (2011). Inflammatory cytokine expression is associated with chikungunya virus resolution and symptom severity. PLoS Negl Trop Dis.

[25] Hoong C, Amin M, Tan T (2021). Viral arthralgia a new manifestation of COVID-19 infection? A cohort study of COVID-19-associated musculoskeletal symptoms. Int J Infect Dis.

[26] Maezawa K, Nozawa M, Sano K (2022). Effects of social isolation associated with the COVID-19 pandemic on hip muscle strength, hip joint pain, and walking ability in patients with osteoarthritis of the hip joint. Geriatr Nurs.

[27] Elings J, Hoogeboom T, Sluis GVD (2015). What preoperative patient-related factors predict inpatient recovery of physical functioning and length of stay after total hip arthroplasty? a systematic review. Clin Rehabil.

[28] Rele S, Dowsey MM, Choong PFM (2020). In pursuit of enhanced recovery after total joint replacement: a narrative review of drivers of length of stay. ANZ J Surg.

[29] Wang L, He W, Yu X (2020). Coronavirus disease 2019 in elderly patients: characteristics and prognostic factors based on 4-week follow-up. J Infect.

[30] Wanhella KJ, Fernandez-Patron C (2022). Biomarkers of ageing and frailty may predict COVID-19 severity. Ageing Res Rev.

[31] Migliorini F, Giorgino R, Hildebrand F (2021). Fragility fractures: risk factors and management in the elderly. Medicina (Kaunas).

[32] Moore MR, Lygrisse KA, Singh V (2022). The effect of femoral head size on groin pain in total hip arthroplasty. J Arthroplast.

[33] Bin Nasser A, Beaulé PE, O’neill M (2010). Incidence of groin pain after metal-on-metal hip resurfacing. Clin Orthop Relat Res.

[34] Lenartowicz KA, Wyles CC, Carlson SW (2023). Prevalence of groin pain after primary dual-mobility total hip arthroplasty. Hip Int.

[35] Götz JS, Benditz A, Reinhard J (2021). Influence of anxiety/depression, age, gender and ASA on 1-Year follow-up outcomes following total hip and knee arthroplasty in 5447 patients. J Clin Med.

[36] Schindler M, Schmitz S, Reinhard J (2022). Pain course after total knee arthroplasty within a standardized pain management concept: a prospective observational study. J Clin Med.

[37] Greimel F, Dittrich G, Schwarz T (2018). Course of pain after total hip arthroplasty within a standardized pain management concept: a prospective study examining influence, correlation, and outcome of postoperative pain on 103 consecutive patients. Arch Orthop Trauma Surg.

[38] Kazarian GS, Anthony CA, Lawrie CM (2021). The impact of psychological factors and their treatment on the results of total knee arthroplasty. J Bone Joint Surg Am.

[39] Luo ZY, Li LL, Wang D (2019). Preoperative sleep quality affects postoperative pain and function after total joint arthroplasty: a prospective cohort study. J Orthop Surg Res.

[40] Reinhard J, Schindler M, Leiss F (2023). No clinically significant difference in postoperative pain and side effects comparing conventional and enhanced recovery total hip arthroplasty with early mobilization. Arch Orthop Trauma Surg.

[41] Benditz A, Maderbacher G, Zeman F (2017). Postoperative pain and patient satisfaction are not influenced by daytime and duration of knee and hip arthroplasty: a prospective cohort study. Arch Orthop Trauma Surg.

[42] Guan W-J, Ni Z-Y, Hu Y (2020). Clinical characteristics of coronavirus disease 2019 in China. New England J Med.

[43] Mahase E (2020). Covid-19: What do we know about “long covid”?. BMJ.

[44] Maclure M (1991). The case-crossover design: a method for studying transient effects on the risk of acute events. Am J Epidemiol.

