# Supplementary material for: Groin pain aggravated in short term contracted by COVID-19 in THA patients: a case-crossover study
Source: J Orthop Surg Res. 2024 Jun 24;19:374. doi: 10.1186/s13018-024-04862-1 (PMC11194934; doi:10.1186/s13018-024-04862-1)
Supplement: Supplementary file 1 — Short-standardized questionnaire about groin pain. [file 13018_2024_4862_MOESM1_ESM.pdf]

Medical ID ______ Name ______ Gender ______ Age ______

1、Have you infected with SARS-COV-2 ?

A. Yes B. No

2、When the infection was comfirmed by the COVID-19 test ?

____

3、How did you treat your SARS-COV-2 infection?

A. Symptomatic treatment (physical cooling, NSAID or cough expectorant drugs)

B. Symptomatic and anti-virals drug (ribavirin, molnupiravir, lopinavir, ritonavir, favipiravir, etc)

C. Symptomatic treatment, anti-virals drug and conbine with hormones or immunosuppressants

4、How about the outcomes of treatment?

A. Rehabilitation B. Becoming septic or admitting to ICU C. Death

5、Have you ever experienced an increase in groin pain?

A. Yes B. No

6、When did the increased groin pain occur?

A. Before COVID-19 B. After COVID-19

7、How long did the increased pain last for? (record the timing and duration of pain aggravation until it returned to baseline levels)

____

8、Is the pain caused by any of the following?

A. Trauma B. Contusion C. Wound infection D. Iliopsoas impingement

E. Inguinal hernia F. Skin disorders G. Cancer metastases H. None of all
